# Feasibility and preliminary evaluation of internet-based compassion and cognitive–behavioral stress-management courses for health care professionals: A randomized controlled pilot trial

**DOI:** 10.1016/j.invent.2022.100574

**Published:** 2022-09-21

**Authors:** Maude Johansson, David Marcusson-Clavertz, Cecilia Gunnarsson, Ida Olsson, Viktor Kaldo, Anna Bratt

**Affiliations:** a Department of Psychology, Faculty of Health and Life Sciences, Linnaeus University, Växjö, Sweden; b Previa, Växjö, Sweden; c Centre for Psychiatry Research, Department of Clinical Neuroscience, Karolinska Institutet, Stockholm Health Care Services, Region Stockholm, Sweden

**Keywords:** Internet-based interventions, Health care professionals, Compassionate mind training course, Cognitive behavioral therapy, Stress management course, Stress of conscience

## Abstract

Health care professionals (HCPs) are exposed to excessive demands in their work environment. In Sweden, work-related stress is one of the most common reasons for sick leaves. Finding cost-effective and easily accessible interventions for HCPs is crucial to counteract stress-related problems and reduce the number of sick leaves. The study aimed to evaluate the feasibility of two internet-based stress management courses and their preliminary effectiveness to reduce HCPs' stress of conscience and work-related stress, and act as a pilot for a larger randomized controlled trial (RCT). Thirty-two HCPs registered for the courses and were randomized to either an internet-based compassion course, ICOP (*n* = 18), or an internet-based cognitive–behavioral course, ICB (*n* = 14). Participants completed measures pre- (i.e., baseline, *n* = 32), post-intervention (at five weeks, *n* = 21), and at follow-up at 10 weeks (*n* = 17), 15 weeks (*n* = 13), and six months (*n* = 12). The study used the following scales: Stress of Conscience Questionnaires, Copenhagen Psychosocial Questionnaire, Self-Compassion Scale, and Professional Quality of Life Scale. Adherence of HCPs (*n* = 21) was measured using the number of logins, messages between course leaders and HCPs, and completed modules. Twelve interviews were conducted to explore participants' perceptions of the accessibility of the courses. Participants reported overall satisfaction with both the ICOP and ICB courses, stating that the courses contributed to new knowledge, individual insight, and behavior change. Both courses showed similar patterns of adherence. Quantitative analyses on pre-and post-intervention data (*n* = 21) showed that stress of conscience and secondary traumatic stress decreased, and self-compassion increased following ICOP. Following ICB, HCPs reported decreased burnout symptoms (according to one of two questionnaires) and increased compassion satisfaction. Both courses seemed feasible, showed promising results, and could be further evaluated in a larger study with a similar design.

## Highlights

• Accessible stress-reduction interventions for health care professionals are needed.
• Online stress management courses show high feasibility.
• This RCT was on internet-based compassion and cognitive–behavioral (ICB) courses.
• The compassion course increased self-compassion and reduced stress of conscience.
• Burnout symptoms reduced after ICB.

## 1 Introduction

Since 2000, health care professionals (HCPs) in Sweden have increasingly taken sick leave owing to work-related stress (Försäkringskassan 2020, n.d.). To the best of our knowledge, this is the first pilot randomized controlled trial (RCT) investigating the feasibility of a compassion course on HCPs' levels of stress of conscience and work-related stress to prevent sick-leave in HCPs.

Sick leaves are associated with a high workload (Bakker and Demerouti, 2017), psychosocial factors, and symptoms of fatigue (Bakker and Demerouti, 2017; Duijts et al., 2007). An increased number of HCPs on sick leave may result in a shortage of actively working HCPs, increasing their work-related stress and preventing them from finding time to meet patients' physical and psychological needs. International figures about sick leave vary across countries. Several reliable studies from Australia, the US, and countries in Europe suggest that stress altogether accounts for approximately 30 % of all work-related illnesses annually (Hoel et al., 2001). When HCPs are unable to practice their profession according to their ethical values, and when their high workload affects their private life, they may experience “stress of conscience,” which is stress related to a troubled conscience in health care, and feelings of guilt (Åhlin et al., 2015; Glasberg, 2007; Glasberg et al., 2006). Stress of conscience has been reported to be associated with higher levels of burnout and stress-related problems (Åhlin et al., 2015) and negatively affect work satisfaction (Lamiani et al., 2017). In turn, high levels of stress of conscience and work-related stress may block the ability to provide compassionate care. Compassion is a motivation to care for and be cared for and is an essential tool for HCPs in their work.

The social and organizational work environment affects work-related stress and mental health issues in HCPs (Andersen and Westgaard, 2013) and thus, the employer has a key role in the preventive environment- and organization work (Försäkringskassan 2020, n.d.). However, measures related to the work environment are often insufficient, given that individual reactions differ regarding the risk of developing stress-related problems, risk of fatigue symptoms, and long-term sick leave.

Contemporary research on work-related stress focuses on increasing individuals' understanding of the factors or attitudes that affect the their stress level or ill health. Strengthening their resilience and understanding of how stress-related problems develop can influence the their ability to handle stress-related problems affected by the work situation. As stress-related health problems are widespread in HCPs, the interventions need to be cost-effective and offered both preventively and for developed stress-related health problems.

Digitization has opened new possibilities for creating cost-effective treatments, and occupational e-mental health interventions have been found to reduce stress and burnout symptoms in HCPs (Phillips et al., 2019). The systematic review of RCT studies by Phillips et al. (2019) found moderate treatment effects on stress (Hedges' g = 0.54) and burnout (g = 0.51).

Compassionate mind training (CMT) is an intervention developed for lowering shame and self-criticism (Gilbert, 2010, Gilbert, 2014), feelings common when experiencing stress of conscience. CMT has been found to increase HCP's abilities to support themselves (self-compassion), lessen their self-criticism, and help them tolerate distressing emotions (Beaumont et al., 2016). McVicar et al. (2021) and McEwan et al. (2020) found that burnout scores decrease after CMT, whereas compassion satisfaction related to work and levels of well-being increase. A number of internet-based CMT interventions have shown promising results regarding increasing self-compassion and reducing depression, anxiety (Kelman et al., 2018; Krieger et al., 2019), and stress (Finlay-Jones et al., 2017). However, we found no internet-based CMT interventions specifically designed for HCPs.

Although work-related stress and stress of conscience are related to anxiety, depression, fatigue, and long-term sick leave, employers have few guidelines for treating work-related stress (Stratton et al., 2017), especially in Sweden. Effective interventions to prevent and reduce work-related stress are important for public health (Björk et al., 2018) as work-related stress often leads to long-term sick leave. Indeed, it is relatively common that those affected do not return to the labor market. Internet-based interventions can be a cost-effective complement to “occupational health care” and “the Health Centre's” efforts to prevent and treat stress-related work stress.

We conducted a feasibility study of two internet-based stress-management courses for HCPs to strengthen their ability to handle internal demands (stress of conscience, self-criticism, and stress) and external work demands (work-related stress) and to reduce the risk of burnout and sick leave. The first was an internet-based compassion course (ICOP) based on CMT, and the second was an internet cognitive–behavioral stress management course (ICB).

### 1.1 Aims

Our primary aim was to assess the feasibility of an internet-based stress management course and explore HCPs' perceptions of such feasibility. We used HCPs' adherence to and acceptability of the course as the primary outcomes of interest. We also examined whether reduced stress of conscience and work-related stress, along with increased self-compassion and professional quality of life, were observed during the interventions.

We formulated the following research questions:

1) How do HCPs assess the feasibility, in terms of adherence and acceptability of each respective course?
2) Do ICOP and ICB courses reduce stress of conscience and work-related stress and increase self-compassion and professional quality of life for HCPs?
3) How large are the preliminary differences between the two courses on a range of outcomes, and how many participants need to be included in a full-scale RCT to evaluate these differences thoroughly?

## 2 Materials and methods

### 2.1 Study design

Our feasibility study considered two five-module internet-based stress management courses for HCPs, namely, ICOP and ICB. We used a randomized design to avoid selection bias in the recruitment to the two interventions and to estimate differences in outcomes, in order to calculate the power needed to evaluate those differences more thoroughly in a future RCT. Our primary hypothesis was that the ICOP course will decrease stress of conscience in HCPs to a greater extent than the ICB course at post-treatment.

The secondary hypotheses were that the ICOP course will have the following effects: increase the professional quality of life (to a greater extent than the ICBT coursed increase self-compassion to a greater extent than course) post-treatment courses.

### 2.2 Recruitment, inclusion criteria, randomization

The recruitment of HCPs took place either through employers in municipalities and regions in Sweden or by advertising on social media (Facebook). Those interested obtained additional information about the study and signed up for it through a website. The inclusion criteria were as follows: full- or part-time work with patients and personal experience of work-related stress. We did not set exclusion criteria.

**Fig. 1:**
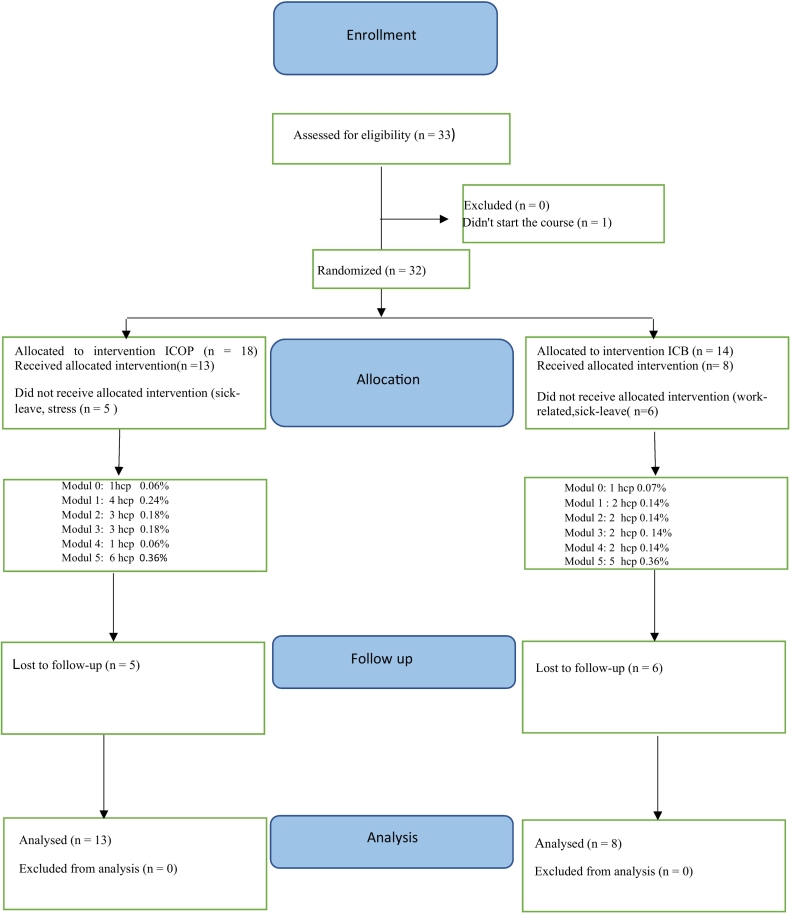
Shows the flowchart of participant enrollment.

In terms of professions, participants included nurses, psychologists, psychotherapists, counsellors, occupational therapists, and doctors. They were randomly assigned into blocks of 20 participants and then 12 participants. An external party (an employee within Region Västra Götaland) used www.random.org to randomize the participants to either ICOP or ICB (See Fig. 1).

### 2.3 Interventions

The courses included five modules, consisting of text messages, video clips, and sound recordings, together with reflective questions and exercises that participants answered in writing. The two courses were designed for HCPs, but only the ICB course was evaluated for stress management. Each module took about an hour to complete, excluding the time needed for weekly homework. Participants completed one module and submitted one homework assignment each week. The course leader gave feedback on participants' homework once a week and was available for questions if needed during the week. Course leaders included licensed psychologists (*n* = 3, one working on ICOP, one on ICB, and one working on both courses) and psychology students (*n* = 6, 4 working on ICOP and 2 on ICB). All the psychology students worked under supervision. All course leaders followed a treatment manual (one for each course) that included the content of all modules and guidelines for participant contact. One course leader specialized in compassion and trained the other course leaders in compassion intervention. All course leaders had experiences and education in ICB.

We measured participants' engagement in the courses by counting the number of modules completed and by confirming their progress through the materials and submission of homework assignments. Participants submitted homework assignments weekly, after each module except Module 5, which had no homework assignments. We did not measure the time taken to complete the homework. The course leaders estimated that answering the assignments and giving feedback took about 10 min.

### 2.4 Internet-based compassion course (ICOP)

The ICOP course is based on Gilbert's compassion-focused therapy, which includes CMT (Gilbert, 2009, Gilbert, 2010, Gilbert, 2014). The course includes psychoeducation on the brain and how it regulates emotion, as well as experiential exercises aimed at increasing the abilities to soothe and care for themselves, developing self-compassion, and reducing self-criticism (see Supplementary material).

### 2.5 Internet-based cognitive–behavioral course (ICB)

The ICB course is partly based on a cognitive behavioral therapy intervention developed for stress-related health problems at Kompetenscentrum psykisk ohälsa, Region Stockholm (https://www.kompetenscentrumpsykiskohalsa.se/material-vuxna2/behandling/stress/; Lindsäter et al., 2018). The original intervention includes 12 sessions, which we reduced to five sessions. The intervention we used included psychoeducation about stress, behavioral exercises, cognitive restructuring, and relaxation techniques (see Supplementary material).

The study was approved by the Swedish Ethical Review Authority (Dnr 2020–05505, Dnr 2021–03415). Data collection occurred between February and May 2021.

### 2.6 Measures

The study included qualitative measures and phone interviews regarding acceptability, helpfulness, and suggested improvements, as well as quantitative measures of credibility, adherence, and preliminary effectiveness of the courses. Internal consistency was evaluated with Cronbach’s α at baseline for each questionnaire.

#### 2.6.1 Phone interview

We conducted semi-structured phone interviews to explore participants' views on the acceptability of the course content, what they found helpful or not helpful and whether the content needed any improvements (see Supplementary for interview guide). We conducted 12 interviews (ICOP = 7, ICB = 5) each lasting 15–30 min.

All participants who received the intervention provided feedback in written form. During the intervention, we asked a number of participants to participate in an interview. When a sufficient number (*n* = 12) had accepted, we stopped asking the rest of the participants.

#### 2.6.2 Adherence

Adherence was measured through the number of times participants logged into the course, the number of messages sent between them and the course leaders, and the number of completed modules.

#### 2.6.3 Treatment credibility scale (TCS)

We used the TCS to assess HCPs' views on the credibility of and expectations regarding the courses (Neff, 2003). Twenty-one HCPs completed the TCS. A mistake in data collection resulted in questions 2 and 4 needing to be excluded. The five-item scale uses a 10-point Likert-type format ranging from 1 (*Not logical at all*) to 10 (*Very logical*). The scale score was the mean of items 1, 3, and 5, which were measured post-intervention (α = 0.90).

#### 2.6.4 Stress of Conscience Questionnaire (SCQ)

This instrument consists of nine items describing various healthcare situations, each made up of two parts, an A question and a B question. Question A concerns how frequently the subject estimates that the situation discussed arises in the workplace. For each A question, there is a subsequent B question in which the degree to which one's conscience is troubled by the given situation is estimated on a 10 cm visual analogue scale (Glasberg et al., 2006; Åhlin et al., 2015). The scale ranges from 0 (*No, it gives me no troubled conscience at all*) to 5 (*Yes, it gives me a very troubled conscience*). Internal consistency was high (α = 0.84).

#### 2.6.5 Copenhagen Psychosocial Questionnaire (COPSOQ)

This instrument examines psychosocial factors at work such as stress and the well-being of employees. It includes 33 questions distributed across 11 scales that reflect either work-related stress or general mental health (Berthelsen et al., 2020; Thorsen and Bjorner, 2010). The questions were assessed on a five-point Likert scale, measuring either intensity (from *To a very large extent* to *To a very small extent*) or frequency (from *Always* to *Never/hardly ever*). Three scales were analyzed, sleep disturbances (α = 0.85), stress symptoms (α = 0.69), and burnout symptoms (α = 0.66).

#### 2.6.6 Self-compassion Scale (SCS)

This instrument includes questions about whether the individuals are understanding rather than self-critical toward themselves when facing difficulties. The 26-item SCS is assessed on a 5-point Likert scale, where 0 corresponds to ‘Almost never’ and 5 to ‘Almost always’. Internal consistency was high (α = 92).

#### 2.6.7 Professional quality of life scale (ProQOL 5)

This instrument includes three scales including 10 items each, assessing both positive and negative aspects of HCP's job, and influence on their professional quality of life (Stamm, 2010; Keesler and Fukui, 2020). The ProQOL 5 consists of 30 questions assessed on a five-point Likert scale, where 0 = *Never* and 5 = *Very often*. Compassion satisfaction (α = 0.89), burnout (α = 0.74), and secondary traumatic stress (α = 0.73) exhibited high internal consistencies.

## 3 Analyses

### 3.1 Qualitative analysis of phone interviews

We analyzed the interview data using thematic analysis including research triangulation to strengthen the credibility of the qualitative analysis (Braun and Clarke, 2021) by authors MJ, CG, and AB. The goal of thematic analysis is to identify themes in the data that are important and use these to address the subject. Data were analyzed according to the six-phase guide of Braun and Clarke (2021). We identified the main themes and subthemes with representative quotes presented in the results section.

### 3.2 Quantitative analysis

We used Jamovi v. 2.2.5 to analyze the data and set α to 0.05. To examine the preliminary effectiveness of the courses, paired samples *t*-tests were performed on each course separately to assess changes from pre- to post-intervention on eight different outcomes. These analyses included participants who completed both pre- and post-intervention assessments (i.e., complete cases). The eight outcomes included stress of conscience, self-compassion, compassion satisfaction, burnout (assessed with two different scales: ProQOL and COPSOQ), secondary traumatic stress, stress symptoms, and sleep disturbances. In addition to these *t*-tests on complete cases, we conducted mixed model analyses that included all participants and time points (i.e., intention-to-treat analyses). Furthermore, to compare the courses on these eight outcomes, repeated measures ANOVAs were performed with Course (ICOP vs. ICB) as a between-subjects factor and Time (pre vs. post) as a within-subjects factor (i.e., complete cases). These tests using only the pre vs. post data were performed to assess the immediate effect of the interventions. The estimated effect sizes for the Course × Time interaction terms were then used as target effect sizes for power analyses using G*power 3.1.9.7. In addition to the repeated measure ANOVA on complete cases, we conducted mixed model analyses that included all participants and time points (pre, post, follow up 1, follow up 2, follow up 3).

As for the mixed model analyses, we modelled the intercepts as random in all analyses. The random effect for each intercept was significant, suggesting substantial individual differences for each of the eight outcomes. The slope of time was modelled as a fixed effect because of the low number of observations in the dataset, which impeded the computation of random slopes. The mixed model results are reported in detail in the Supplementary material.

## 4 Results

Twenty-one (66 %) out of 32 participants (aged 27–56 years, 88.9 % women) completed both baseline and post-intervention measures (ICOP, *n* = 13, ICB, *n* = 8).

### 4.1 Acceptability

The qualitative analyses of the phone interviews identified two main themes and three subthemes regarding the accessibility of the ICOP and ICB courses: *practicalities and usage* (content, treatment support, obstacles) and *changes and insight*.

#### 4.1.1 “Practicalities and usage”

##### 4.1.1.1 Content

Several participants mentioned that the content was understandable and helped them appreciate the psychoeducational elements, which led to an increased understanding. The exercises and homework in both courses were appreciated. Most of the informants were satisfied with the mixture of text, moving material, and graphic images. Regarding the ICOP course, one informant mentioned that some exercises were tough, such as difficulties with imagination or finding examples. One ICB informant did not think that the course content was adapted for nursing staff.

Overall, the informants from both courses were satisfied with the five modules over the five-week format. Most of the informants were satisfied with the amount of text in the ICB course in contrast to the ICOP course, which several participants found to be too text heavy.

"I feel that it was a lot of work, requiring a lot of thinking and how to do [the exercises], … and how do I think and how do I reflect and in what situations and such. It's not just like reading". (Participant in the ICOP course).

Some participants in the ICB course appreciated that the course was internet-based, which facilitated greater accessibility and reduced stress.

##### 4.1.1.2 Treatment support

Treatment support referred to support from the course leader, such as written responses to participants' messages, reflections, and submitted homework. Informants from both courses mentioned the importance of receiving support and feedback from their course leader. For some informants, receiving support motivated them to continue the course. However, informants in both courses wanted more contact during the course, such as regular telephone contact. According to a participant in the ICOP course: "He wrote nice answers and I felt a little seen and such".

##### 4.1.1.3 Obstacles

Some informants from both courses mentioned technical difficulties, and a few expressed dissatisfactions with the login to the platform, the two-step authentication. One participant described repeatedly forgetting the code, which meant that the participant only logged in once a week and, therefore, also forgot the course content. Another participant described having two telephone numbers and seldom having the correct telephone with which the code was sent, which affected accessibility.

#### 4.1.2 “Changes and insight”

Informants in both courses expressed that they gained new knowledge, reflections, and insights, which then led to behavioral changes. Some informants mentioned forming increased ability to set boundaries for themselves and increased self-care abilities. One ICB informant mentioned improved self-assertive communication. Several informants mentioned improving their recovery behavior. Informants in both courses described gaining new insights and the ability to live life according to one's values, which, for one participant, led to a change in jobs and to another, an interrupted change in jobs. They realized that “work is not everything.”

“It has made me start going home on time more often. I have started to say no when someone asks me to do things beyond what I feel I have time for. I have withdrawn my resignation because I feel that I have a chance to be there and still feel good. "I have changed my attitude in how I handle my job". (Participant in the ICB course).

The ICOP course included lessons and insights on the function of self-criticism and importance of being kind to oneself but also looking after one's values and lowering one's demands. ICOP informants expressed that their self-criticism decreased while their self-compassion and acceptance increased. Two ICOP informants noticed that compassion for others had also increased. Moreover, some informants mentioned that the ICB course provided new insights regarding what was stressful and the importance of recovery. An ICOP informant also mentioned improved sleep, and most informants described an increased kindness toward themselves.

I feel calmer when I'm at work and if I feel stressed, it's easier for me to calm down. (Participant in the ICOP course).

### 4.2 Treatment adherence

Two HCPs (1 ICOP, 1 ICB) dropped out of treatment immediately after the start. Four HCPs (2 ICOP, 2 ICB) completed one module and then dropped out. Two HCPs (ICB) dropped out after module 2. Twenty-one participants completed the two courses. We conducted independent samples *t*-tests that showed no significant differences between courses (*p* = 0.18–0.24) regarding number of times participants were logged in during the ICOP (*M* = 14.8, *SD* = 8.5) and ICB (*M* = 13.3, *SD* = 10.4) courses; number of messages submitted during the ICOP (*M* = 8.2, *SD* = 4.6) and ICB (*M* = 11.3, *SD* = 7.4) courses; and number of completed modules for the ICOP (*M* = 2.9, *SD* = 1.7) and ICB (*M* = 3.0, *SD* = 2.3) courses.

### 4.3 Treatment credibility

We used the TCS, measured at the post-intervention assessment, to evaluate the credibility of the two courses. Mean credibility scores were high for both ICOP (*M* = 7.15, *SD* = 2.04, *n* = 13) and ICB (*M* = 7.58, *SD* = 1.60, *n* = 8). All participants provided mean ratings above 4.66 except one potential outlier, who gave the ICOP course the minimum rating of 1 on the 1–10 scale. We found no significant difference between the courses, *t*(19) = −0.51, *d* = −0.23; *d* = 0.07, with the outlier excluded.

### 4.4 Missing data

Table 1 gives a descriptive summary of all eight outcomes of interest. For this feasibility trial, 21 (66 %) out of 32 participants completed both the pre- and post- intervention assessments of all quantitative questionnaires. Those who completed the post-intervention assessment did not significantly differ from those who failed to complete it on any of the eight outcomes at the pre-intervention assessment (*t*(30) ≤ 1.08, *p* ≥ 0.290, Cohen’s *d* < 0.40, see Supplementary material). As shown in Table 1, the study had additional attrition throughout the three follow-up assessments. To evaluate this attrition, we compared the 12 participants who completed the final assessment with the other 20 participants who failed to complete it by using the last observed assessment for each person (i.e., “last observation carried forward”). The differences between these groups were not significant, *t*(30) ≤ 1.84, *p* ≥ 0.075, Cohen’s *d* < 0.67, see Supplementary material) Nonetheless, burnout, sleep disturbance, and secondary traumatic stress symptoms were moderately higher among those who did not complete the final assessment (Cohen’s *d*s ≥ 0.50).

**Table 1:** Descriptive summary of research measures across courses.

Variable	Time	Course							
		ICOP				ICB			
		N	M	SD	Skew	N	M	SD	Skew
Stress of conscience (SCQ)	Pre	18	73.91	36.37	−0.1	14	73.92	40.56	0.03
	Post	13	56.65	39.1	0.53	8	73.38	40.13	−0.64
	FU1	9	30.23	27.93	1.31	8	61.51	45.36	−0.18
	FU2	5	29.13	34.57	0.96	7	71.29	43.63	−0.22
	FU3	7	43.09	35.25	0.33	5	74.59	42.36	−1.31
Self-compassion (SCS)	Pre	18	2.76	0.55	−0.66	14	2.78	0.74	0.60
	Post	13	3.20	0.59	−1.95	8	3.06	0.54	0.68
	FU1	9	3.40	0.7	−0.40	8	3.11	0.56	−0.24
	FU2	5	3.94	0.42	0.49	7	3.23	0.73	0.57
	FU3	7	3.65	0.48	−0.43	5	2.90	0.48	0.20
Compassion satisfaction (PROQOL)	Pre	18	34.83	5.52	−2.00	14	34.21	6.81	−0.19
	Post	13	36.85	5.91	−0.56	8	36.38	6.93	0.59
	FU1	9	39.00	5.41	0.05	8	34.00	7.05	−0.03
	FU2	6	42.17	6.31	−0.20	7	34.86	7.06	−0.15
	FU3	7	36.86	5.73	0.55	5	35.40	5.77	0.14
Burnout (PROQOL)	Pre	18	27.61	4.57	0.13	14	28.64	5.14	−0.55
	Post	13	25.85	4.71	0.56	8	25.75	5.97	−0.30
	FU1	9	22.89	4.73	0.00	8	25.75	4.92	−0.27
	FU2	6	19.83	5.27	−0.2	7	25.00	6.3	0.00
	FU3	7	22.43	4.54	−0.75	5	23.80	4.02	−0.45
Secondary traumatic stress (PROQOL)	Pre	18	23.50	4.59	0.60	14	23.64	5.3	0.33
	Post	13	21.62	4.86	1.24	8	24.63	5.85	−0.08
	FU1	9	19.67	3.77	0.15	8	22.50	4.96	−0.64
	FU2	6	18.50	3.67	−0.82	7	20.57	6.9	0.33
	FU3	7	18.57	4.39	1.13	5	21.20	3.27	0.42
Stress symptoms (COPSOQ)	Pre	18	64.24	11.9	0.47	14	61.16	18.7	−0.12
	Post	13	58.65	17.22	−0.33	8	62.50	21.39	0.46
	FU1	9	51.39	25.92	0.46	8	57.81	25.17	0.23
	FU2	5	50.00	37.24	0.04	7	52.68	24.96	−0.55
	FU3	7	53.57	29.94	−0.29	5	55.00	24.76	−0.60
Burnout symptoms (COPSOQ)	Pre	18	61.11	14	−0.26	14	60.71	17.73	−0.88
	Post	13	50.64	19.38	0.27	8	61.46	12.55	0.15
	FU1	9	34.26	24.1	0.30	8	54.17	19.42	−0.90
	FU2	5	31.67	21.57	−0.50	7	53.57	23.99	−0.01
	FU3	7	40.48	18.9	0.85	5	51.67	21.57	−0.36
Sleep disturbances (COPSOQ)	Pre	18	48.26	19.03	0.96	14	51.79	28.32	−0.18
	Post	13	35.10	22.18	0.18	8	40.63	27.95	−0.22
	FU1	9	29.86	31.68	1.54	8	37.50	32.04	0.28
	FU2	5	32.50	39.13	1.84	7	34.82	25.99	−0.06
	FU3	7	28.57	34.21	1.88	5	35.00	35.52	0.48

*Note*. ICOP = internet-based compassion course. ICB = internet-based cognitive–behavioral course. SCQ = Stress of Conscience Questionnaires. SCS = Self-Compassion scale. PROQOL = Professional Quality of Life scale. COPSOQ = Copenhagen Psychosocial Questionnaire. FU1 = 10 weeks post-intervention, FU2 = 15 weeks post-intervention, FU3 = six months post-intervention.

### 4.5 Preliminary results on the effectiveness of the course

Table 2 shows the results of the paired samples *t*-tests assessing changes from pre- to post-intervention assessments. Participants who took the ICOP course reported significantly reduced stress of conscience and secondary traumatic stress and increased self-compassion post-intervention (*n* = 13). Participants who took the ICB course reported significantly reduced burnout (ProQOL) and increased compassion satisfaction post-intervention (*n* = 8). Owing to the small sample size, some of the non-significant results included medium effect size estimates (Cohen’s *d* > 0.50), including reduced burnout symptoms (COPSOQ) and sleep disturbances following ICOP, and increased self-compassion and reduced sleep disturbances following ICB. Although most scales showed acceptable reliability in this study, it is worth noting that the burnout symptoms subscale exhibited moderate reliability.

**Table 2:** Paired samples *t*-tests on the preliminary effectiveness of the courses (pre vs. post, each course analyzed separately).

Outcome	Course							
	ICOP (*n* = 13)				ICB (n = 8)			
	*t*(12)	*p*	*d*	95 % *CI*	*t*(7)	*p*	*d*	95 % *CI*
Stress of conscience (SCQ)	−3.09	0.009**	−0.86	[−1.49, −0.20]	−0.70	0.504	−0.25	[−0.95, 0.46]
Self-compassion (SCS)	4.25	0.001**	1.18	[0.45, 1.88]	1.82	0.112	0.64	[−0.14, 1.39]
Compassion satisfaction (ProQOL)	1.13	0.283	0.31	[−0.25, 0.86]	3.67	0.008**	1.30	[0.31, 2.24]
Burnout (ProQOL)	−1.41	0.184	−0.39	[−0.95, 0.18]	−3.72	0.007**	−1.31	[−2.26, −0.32]
Secondary traumatic stress (ProQOL)	−2.38	0.035*	−0.66	[−1.25, −0.05]	−0.10	0.922	−0.04	[−0.73, 0.66]
Stress symptoms (COPSOQ)	−1.43	0.178	−0.40	[−0.96, 0.18]	0.33	0.749	0.12	[−0.58, 0.81]
Burnout symptoms (COPSOQ)	−1.90	0.082	−0.53	[−1.10, 0.07]	0.00	1.00	0.00	[−0.69, 0.69]
Sleep disturbances (COPSOQ)	−2.04	0.064	−0.57	[−1.14, 0.03]	−2.20	0.064	−0.78	[−1.56, 0.04]

*Note*. ICOP = internet-based compassion course. ICB = internet-based cognitive–behavioral course. SCQ = Stress of Conscience Questionnaires. SCS = Self-Compassion scale. ProQOL = Professional Quality of Life scale. COPSOQ = Copenhagen Psychosocial Questionnaire. **p < .01; *p < .05.

The mixed model intention-to-treat analyses with all participants and five time points yielded largely similar results (see Supplementary material). As for the ICOP course (*n* = 18), the results indicated that stress of conscience, secondary traumatic stress, and burnout symptoms (COPSOQ) were significantly reduced, and self-compassion was significantly increased, at the post-intervention assessment compared with the pre-intervention assessment. These remained significantly different from the pre-intervention assessment across all three follow- up assessments. As for the ICB course (*n* = 14), the results indicated that burnout (ProQOL) was significantly reduced at the post-intervention assessment and remained significantly reduced across all three follow-up assessments, whereas self-compassion was significantly increased at the post-intervention assessment and the first two follow-up assessments, but not the third follow-up.

### 4.6 Estimated differences between courses and number of participants needed in future RCT

The repeated measures ANOVAs with Course (ICOP vs. ICB) as a between-subjects factor and Time (pre vs. post) as a within-subjects factor did not reveal any significant two-way interaction on any of the eight outcomes (η_p_^2^ < 0.12, *p* > 0.130, see Supplementary material). That is, the effect of the intervention did not significantly depend on whether participants completed an ICOP or ICB course, although this pilot sample was not powered to detect such effects.

The observed effect sizes for the Course × Time interaction terms were then used as targets to estimate the number of participants needed for future RCTs. The primary outcome of interest was stress of conscience and, based on this trial, a sample of 100 participants in each arm would be needed to obtain over 90 % power for detecting such effect as observed here (see Supplementary material).

## 5 Discussion

This study explored the feasibility, acceptability, and adherence of two internet-based stress management courses for HCPs. We also investigated the possible effects of internet-based stress management courses on stress of conscience, self-compassion, and professional quality of life. In general, the participating HCPs were satisfied with both the ICOP and ICB courses. They appreciated the course content and treatment support as well as reported behavior changes that positively affected them. Treatment adherence showed no difference between the courses. Dropouts were most common early in the treatment but the mean credibility scores were high for both courses. These results support the feasibility of the study.

The preliminary results revealed that HCPs who performed the ICOP course reported significantly reduced stress of conscience and secondary traumatic stress and increased self-compassion post-intervention. By contrast, participants who completed the ICB course reported significantly reduced burnout (ProQOL) and increased compassion satisfaction post-intervention. The results were consistent with our expectations.

The courses had different focuses, with ICOP focusing on stress of conscience and self-compassion and ICB focusing on work-related stress and burnout. The ICB course may increase HCPs´ abilities to recover from stress, whereas the ICOP course may have a more indirect path where HCPs learn how to care for themselves, decrease self-criticism and stress of conscience, and these effects are hypothesized to take longer time, than those for the ICB participants. It may be that the choice of method and how effective it is depend on what it is that affects participant's stress and job satisfaction. ICOP has a greater focus on individuals whose stress comes from within, i.e., who need help with their conscience in order to be able to look adequately at the work situation, whereas ICB may provide better conditions for individuals who need to manage their external stress environment. Both courses were hypothesized to decrease work-related stress to the same degree in the long run. However, the results must be interpreted with caution and need to be investigated in a larger sample. To the best of our knowledge, this is the first pilot RCT investigating the feasibility of a compassion course on HCPs' levels of stress of conscience and work-related stress. Meanwhile, ICB interventions are well proven for reducing stress (Heber et al., 2017).

Our results are promising and consistent with earlier findings on compassion interventions for HCPs, which have shown increased self-compassion (Beaumont et al., 2016; McEwan et al., 2020). Based on this trial, future RCTs would require about 100 participants per course to obtain over 90 % power to detect differences between ICOP and ICB in stress of conscience. Such sample size would also exhibit over 90 % power to detect differences in self-compassion, compassion satisfaction, burnout, and stress symptoms, based on the effect size estimates of this trial.

Only two-thirds of the participants completed both pre-and-post intervention measures, and additional attrition occurred in the follow-ups. This is consistent with other reports showing that internet treatment participants often fail to complete the entire treatment and have a high dropout for completing assessments (Carlbring et al., 2006; Melville et al., 2010). Dropouts are also frequent in face-to-face treatment interventions. Despite prompt research on client attrition from therapy, researchers remain ignorant of the differences between obstacles and success to engage and retain clients in therapy (Barrett et al., 2009). Most dropouts in both our stress management courses ocurred early before the start of the course or after the first module, an indication that participants completing one module are more likely to complete the entire course. Reducing the number of dropouts may require the examination of participants' motivation and ability to complete the course according to plan before the course starts, to avoid participants without prerequisites or motivation to complete the course. Treatment dropout is often perceived as a failure and a negative experience for the participant.

We implemented several adjustments to decrease the number of dropouts for the larger-scale study (see Bratt et al., 2021). For example, before the start of the courses, we added a short interview in which HCPs can address issues that may impact their course adherence. Several participants expressed a preference for phone contact in addition to written communication. Thus, we offered participants one short phone contact per week. Furthermore, the amount of text in the ICOP was reduced considerably, as some participants found the text materials too long. In the future, we will administer a questionnaire to those who drop out of the study in case they want to provide information on their reasons for dropping out. Research reducing dropouts may be as important as that on treatment effectiveness. Indeed, the factors that contribute to the completion of the treatment or contribute to dropouts need to be examined.

### 5.1 Limitations

This feasibility trial included a small sample that was largely composed of women. This sex difference is probably because a majority of HCPs in Sweden are women (Socialstyrelsen, n.d.). The quantitative effects of the two courses could not be discriminated in this study and should be evaluated in a larger sample in which sex differences in responsiveness could be examined as well. Moreover, the trial had a rather large attrition of data at post-treatment owing to the large attrition at follow-ups, which always motivates a more restrictive interpretation of results, although the missing data analyses did not show significant effects attributable to attrition. Another limitation that probably affected adherence to the courses was that the courses were not masked and some participants expressed disappointment when they were not offered the course they desired. A possible explanation of the relatively low adherence might be that some participants were more interested in a specific course as professionals than in work-related stress. In our protocol for the larger trial (Bratt et al., 2021), we altered the design to improve adherence by making adjustments, such as masking the included interventions and setting weekly phone contacts to supplement the written communication with participants and course leaders. Another limitation was the lack of a control group, which we intend to include in our larger trial as a waitlist control group. We will also have a measure of sick leave in our larger trial; this was not possible in this feasibility study owing to time constraints.

### 5.2 Conclusions

This pilot RCT is, to the best of our knowledge, the first study investigating the feasibility of a compassion course on HCPs' levels of stress of conscience. Qualitative data revealed that participants, in general, were satisfied with both courses, which contributed to new knowledge, individual insight, and behavior change. Pre- and post-intervention quantitative data showed a reduction in stress of conscience and secondary traumatic stress and an increase in self-compassion following ICOP. Following ICB, participants reported reduced burnout symptoms (according to one of two questionnaires) and increased compassion satisfaction.

We found similar patterns of adherence to both courses, where 21 participants completed the courses (66 %), others were dropouts, and the three follow-up sessions recorded additional attrition. Several adjustments were made to improve adherence for future larger-scale studies, which would be needed before any conclusions can be drawn regarding the effectiveness of internet-based stress management.

## Declaration of competing interest

The authors declare that they have no known competing financial interests or personal relationships that could have appeared to influence the work reported in this paper.

## Appendix A Supplementary material

Supplementary material Image 1
