# Supplementary material for: Feasibility and preliminary evaluation of internet-based compassion and cognitive–behavioral stress-management courses for health care professionals: A randomized controlled pilot trial
Source: Internet Interv. 2022 Sep 21;30:100574. doi: 10.1016/j.invent.2022.100574 (PMC9520015; doi:10.1016/j.invent.2022.100574)

**Supplementary material for the manuscript:**

**“Feasibility and preliminary evaluation of internet-based compassion and cognitive-behavioural stress-management courses for healthcare professionals: A randomized controlled trial”**

**Semi-structured phone interviews to explore participants’ views on the acceptability of the course content,**

1. Can you describe your experience of the compassion course/stress management course?

2. Did you notice any changes in your approach to your work after the work?

3. Where there any changes for you in how to manage workrelated stress after the course?

4. Did you notice any other changes after the course ? If so, please describe it.

5. Did you notice any other changes how to manage stress or life outside of work?

6. How satisfied or dissatisfied are you with the course?

• In what way?

• What are you satisfied/dissatisfied with?

• In what way can the course be improved?

7. Is there anything else you want to add?

**Descriptions of ICB stress management course**

| Module | Theme |
| --- | --- |
| 1 | **Psychoeducation and Behavior Analyses**  The first step consisted of behavior analysis, and psychoeducation about stress and recovery. The course participants were taught how to carry out behavioral analyzes and record their activity level during the days to identify excess and deficit behavior, thus elucidating which change was necessary. |
| 2 | **Survey**  The behavior analysis continues in module two and with more in-depth information about recovery, which also included e.g. relaxation techniques, sleep and how course participants can work brain-smart. The concept of behavioral activation was introduced at the end of this module. |
| 3 | **Behavioral activation: value-based work**  The next module focused on life values and living a balanced life. The course participants reflected on whether they live according to their values and then formulate goals for a change accordingly. |
| 4 | **Exposure**  In module four, participants worked with exposure, behavioral experiments and ow to break emotion-driven behaviors and find more helpful alternative behaviors. |
| 5 | Exposure  Deepening the exposure-work, and how to break dysfunctional life rules. The focus is cognitive restructuring as part of how to deal with stressful thoughts. Psychoeducation is obtained about thought traps and the course participant gets to identify alternative thoughts and set up and carry out behavioral experiments. Finally, communication skills are also presented to learn to set boundaries. |

**Descriptions for the ICOP stress management course**

| Module | Themes |
| --- | --- |
| 1 | **Compassion and three emotional-regulation systems**  Psychoeducation about compassion,the three emotional-regulation systems, attention and conscious presence. Introducing the self-critical- and compassionate parts. Practice of simple breathing exercises. |
| 2 | **Evolution, the brain and difficult emotions**  Psychoeducation about the old and new brain and how different parts of the brain are connected to the three emotional systems. In this module, the course participant gains an understanding of the function of different emotions. The practice of how to increase the ability to care for onseself is deepend and how to reduce self-criticism and stress. |
| 3 | **Our multiple selves**  The focus is increase the ability to understand and take care of our different parts, our multiple selves, depending on how we feel and react in different situations. The course participant learns strategies for stopping, practicing conscious presence and further practice in creating a compassionate self. |
| 4 | **Take care of yourself when life is hard**  In-depth psychoeducation about the self-critical side and how the compassionate part can care for the inner critic. The participants learn how to balance emotions and take care of themselves when life is difficult. |
| 5 | **Self-compassion**  The last module focused on the compassionate part and the course participant write a wise and caring letter to herself. A concluding section were the course participant plan how to continue the compassion work. |

**Examining attrition at the *post-intervention* assessment**

Out of the 32 participants who completed the pre-intervention assessment, 11 failed to complete the post-intervention assessment, whereas 21 completed it. The main manuscript stated that these two groups did not differ significantly at the pre-intervention assessment on any of the eight outcomes of interest, *t*(30) < 1.08, *p* > .290, *d* < 0.40. Table S1 shows the results for all eight outcomes.

**Table S1**

***Comparisons of those who completed the post-intervention assessment and those who did not using the pre-intervention assessment***

| Variable | Group | | Difference | | |
| --- | --- | --- | --- | --- | --- |
|  | Completed post (*n*=21) | Failed to complete post (*n*=11) |  |  |  |
|  | *M (SD)* | *M (SD)* | *Cohen’s d* | *t*(30) | *p* |
| Stress of Conscience (SCQ) | 79.09 (40.34) | 64.04 (31.15) | 0.40 | 1.08 | .290 |
| Self-compassion (SCS) | 2.74 (0.53) | 2.83 (0.82) | -0.15 | -0.4 | .692 |
| Compassion Satisfaction (PROQOL) | 34.05 (6.77) | 35.55 (4.37) | -0.25 | -0.66 | .513 |
| Burnout (PROQOL) | 28.29 (5.45) | 27.64 (3.29) | 0.13 | 0.36 | .721 |
| Secondary Traumatic Stress (PROQOL) | 23.9 (5.14) | 22.91 (4.35) | 0.20 | 0.55 | .588 |
| Stress symptoms (COPSOQ) | 63.1 (13.96) | 62.5 (17.68) | 0.04 | 0.1 | .917 |
| Burnout Symptoms (COPSOQ) | 60.71 (16.28) | 61.36 (14.56) | -0.04 | -0.11 | .912 |
| Sleep disturbances (COPSOQ) | 48.51 (24.6) | 52.27 (21.15) | -0.16 | -0.43 | .670 |

*Note*. SCQ = Stress of conscience Questionnaires. SCS = Self-compassion Scale. PROQOL = Professional quality of life scale. COPSOQ = Copenhagen Psychosocial Questionnaire.

**Examining attrition at the *follow-up* assessments with independent samples t-tests**

Out of the 21 participants who completed both pre and post-intervention assessments, there were 9 participants who failed to complete the last assessment, follow-up 3. We used the last observed assessment of those 9 participants as a proxy and compared those scores with the follow-up 3 scores of the participants who successfully completed the final assessment (i.e., *last observation carried forward*). Table S2 shows the results. In the manuscript we stated that the groups did not significantly differ on any outcome, but it is notable that burnout, stress, and sleep disturbances were slightly higher in the group that failed to complete the final assessment

**Table S2**

***Comparisons of those who completed the final assessment (FU3) and those who did not (using their last reported assessment as a proxy, i.e., either Post, FU1, or FU2)***

| Variable | Group | | Difference | | |
| --- | --- | --- | --- | --- | --- |
|  | Completed FU3 (*n*=12) | Failed to complete FU3 (*n*=9) |  |  |  |
|  | *M (SD)* | *M (SD)* | *d* | *t*(19) | *p* |
| Stress of Conscience (SCQ) | 56.21 (39.92) | 46.13 (38.94) | -0.26 | -0.58 | .570 |
| Self-compassion (SCS) | 3.34 (0.60) | 3.26 (0.65) | -0.12 | -0.26 | .798 |
| Compassion Satisfaction (PROQOL) | 36.25 (5.53) | 37.89 (5.26) | 0.30 | 0.69 | .501 |
| Burnout (PROQOL) | 23.00 (4.20) | 24.44 (6.37) | 0.28 | 0.63 | .538 |
| Secondary Traumatic Stress (PROQOL) | 19.67 (4.03) | 20.33 (3.94) | 0.17 | 0.38 | .709 |
| Stress symptoms (COPSOQ) | 54.17 (26.69) | 59.03 (19.04) | 0.20 | 0.46 | .648 |
| Burnout Symptoms (COPSOQ) | 45.14 (19.93) | 50.00 (19.98) | 0.24 | 0.55 | .587 |
| Sleep disturbances (COPSOQ) | 31.25 (33.29) | 40.97 (22.99) | 0.33 | 0.75 | .462 |

*Note*. Those who did not complete any post-intervention assessment were excluded from this analysis. SCQ = Stress of conscience Questionnaires. SCS = Self-compassion Scale. PROQOL = Professional quality of life scale. COPSOQ = Copenhagen Psychosocial Questionnaire.

**Examining differences between courses with repeated measures ANOVAs**

To compare the effectiveness of the two courses, repeated measures ANOVAs were performed with Course as the between-subjects factor (ICOP vs. ICB) and Time (pre vs. post) as the within-subjects factor. The main manuscript stated that there were no two-way interaction between Course and Time (see Table S3). There were significant main effects for self-compassion, compassion satisfaction, stress of conscience, sleep disturbances and burnout (measured with the PROQOL, but not COPSOQ).

**Table S3**

***Results from Repeated Measures ANOVAs (N = 21) with Course (ICOP vs ICB.) and Time (Pre vs. Post) as predictors of self-reported outcomes***

| Outcome | Effect | | | | | | | | |  |
| --- | --- | --- | --- | --- | --- | --- | --- | --- | --- | --- |
|  | Time | | | Course | | | Time × Course | | | |
|  | *F* | *P* | η_p_^2^ | *F* | *p* | η_p_^2^ | *F* | *p* | η_p_^2^ | |
| Stress of Conscience (SCQ) | 5.40 | .031 | .22 | 0.37 | .548 | .02 | 1.01 | .327 | .05 | |
| Self-compassion (SCS) | 16.45 | <.001 | .46 | 0.00 | .954 | .00 | 1.63 | .217 | .08 | |
| Compassion Satisfaction (PROQOL) | 9.63 | .006 | .34 | 0.52 | .480 | .03 | 2.5 | .130 | .12 | |
| Burnout (PROQOL) | 10.67 | .004 | .36 | 0.21 | .651 | .01 | 1.81 | .195 | .09 | |
| Secondary Traumatic Stress (PROQOL) | 0.86 | .366 | .04 | 1.19 | .290 | .06 | 0.47 | .500 | .02 | |
| Stress symptoms (COPSOQ) | 0.44 | .514 | .02 | 0.00 | .979 | .00 | 1.34 | .261 | .07 | |
| Burnout Symptoms (COPSOQ) | 1.61 | .219 | .08 | 0.83 | .374 | .04 | 1.61 | .219 | .08 | |
| Sleep disturbances (COPSOQ) | 6.55 | .019 | .26 | 0.15 | .705 | .01 | 0.13 | .719 | .01 | |

*Note*. ICOP = internet-based compassion course. ICB = internet-based cognitive-behavioral course. SCQ = Stress of conscience Questionnaires. SCS = Self-compassion Scale. PROQOL = Professional quality of life scale. COPSOQ = Copenhagen Psychosocial Questionnaire

**Power analyses for future RCTs (ANOVAs; pre vs post)**

The main manuscript stated that 100 participants would be needed in each arm to obtain at least 90% power to the detect the observed effect sizes for stress of conscience. The other seven outcomes of interest were also analyzed. The results of these power analyses are reported in Table S4. These estimates were based on the two-way interaction effects from the repeated measures ANOVAs (see Table S3).

**Table S4**

***Results from Power Analyses for the Course × Time interaction effects based on the Repeated Measures ANOVAs (n = 21) comparing two courses (ICB vs. ICOP) across points (Pre vs. Post)***

| **Outcome** | **η_p_^2^ (SPSS)** | **Effect size f (G*power)** | **Total**  **sample size for 80% power** | **Total**  **sample size for 90% power** |
| --- | --- | --- | --- | --- |
| Stress of Conscience | .051 | .232 | 150 | 200 |
| Self-compassion | .079 | .293 | 96 | 128 |
| Compassion Satisfaction (PROQOL) | .116 | .362 | 64 | 84 |
| Burnout (PROQOL) | .087 | .309 | 88 | 116 |
| Secondary Traumatic Stress (PROQOL) | .024 | .157 | 324 | 432 |
| Stress symptoms (COPSOQ) | .066 | .266 | 116 | 154 |
| Burnout Symptoms (COPSOQ) | .078 | .291 | 98 | 130 |
| Sleep disturbances (COPSOQ) | .007 | .199 | 1118 | 1496 |

**Examining all five time points using Mixed model analyses**

Mixed model analyses were computed using all five time points (pre, post, follow up 1, 2, and 3). These random intercepts analyses were based on the 21 participants who completed at least both the pre- and post-intervention assessments. P-values were not adjusted for multiple analyses (α = .05). Analyses were run in Jamovi v 2.2.5 and a sample syntax is shown below (gamlj package):

gamlj::gamljMixed(

formula = Self-compassion ~ 1 + Course + Time + Course:Time+( 1 | ParticipantId ),

data = data,

contrasts=c(Course = "simple", Time = "polynomial"),

plotHAxis = Time,

plotSepLines = Course,

plotRaw = TRUE,

plotError = "ci",

simpleVariable = Course,

simpleModerator = Time,

lrtRandomEffects = TRUE,

cimethod = "boot",

qq = TRUE,

normTest = TRUE,

normPlot = TRUE,

residPlot = TRUE,

duplicate = 32)

As shown in Table S5, there were significant main effects of Time across all outcomes except stress symptoms, which means that stress of conscience, self-compassion, compassion satisfaction, burnout (measured with both COPSOQ and PROQOL), secondary traumatic stress, and sleep disturbances varied across the five-time points. Follow-up polynomial analyses indicated a significant linear increase in self-compassion and compassion satisfaction and decrease in burnout (PROQOL) and sleep disturbances across time (see Fig S1-8, *n* = 21).

Although this feasibility trial was not powered to detect differences between courses, there were two significant Course × Time interactions on PROQOL burnout and self-compassion. Simple effects follow-ups on burnout at each of the five assessments indicated that ICOP participants reported lower burnout (PROQOL) than ICB participants at follow-up 2, *B* = 6.23, *SE* = 2.56, *p* = .020 (no other time point indicated a significant simple effect, *p* >.178, see Fig S4). Simple effects follow-up on self-compassion did not reveal any significant difference between ICOP and ICB participants at any time point (*p* > .066, see Fig. S2). The reported differences between courses should be interpreted with caution as the sample size was small and *p*-values on follow up analyses were not adjusted.

**Table S5**

***Results from 8 mixed model analyses with Time (pre vs. post vs. FU1. vs. FU2. Vs. FU3) and Course (ICOP vs. ICB) as predictors (n = 21)***

| Outcome | Fixed effect | | |
| --- | --- | --- | --- |
|  | Course | Time | Time × Course |
| Stress of Conscience (SCQ) | *F*(1,19) = 2.33, *p* = .144 | *F*(4,55) = 5.02, *p* = .002****** | *F*(4,55) = 1.45, *p* = .231 |
| Self-compassion (SCS) | *F*(1,19) = 1.14, *p* = .298 | *F*(4,55) = 15.66, *p* < .001******* | *F*(4,55) = 3.43, *p* = .014***** |
| Compassion Satisfaction (PROQOL) | *F*(1,20) = 2.16, *p* = .158 | *F*(4,56) = 3.66, *p* = .010***** | *F*(4,56) = 1.98, *p* = .110 |
| Burnout (PROQOL) | *F*(1,19) = 1.61, *p* = .220 | *F*(4,56) = 11.88, *p* < .001******* | *F*(4,56) = 2.60, *p* = .046***** |
| Secondary Traumatic Stress (PROQOL) | *F*(1,20) = 1.82, *p* = .193 | *F*(4,56) = 8.49, *p* < .001******* | *F*(4,56) = 0.32, *p* = .864 |
| Stress symptoms (COPSOQ) | *F*(1,20) = 0.18, *p* = .674 | *F*(4,56) = 1.18, *p* = .332 | *F*(4,56) = 0.30, *p* = .879 |
| Burnout Symptoms (COPSOQ) | *F*(1,19) = 3.21, *p* = .089 | *F*(4,55) = 4.68, *p* = .003****** | *F*(4,55) = 1.32, *p* = .273 |
| Sleep disturbances (COPSOQ) | *F*(1,20) = 0.32, *p* = .580 | *F*(4,55) = 3.50, *p* = .013***** | *F*(4,55) = 0.20, *p* = .937 |

**Note.** Satterthwaite method for degrees of freedom (rounded to nearest integer values). ICOP = internet-based compassion course. ICB = internet-based cognitive-behavioral course. SCQ = Stress of conscience Questionnaires. SCS = Self-compassion Scale. PROQOL = Professional quality of life scale. COPSOQ = Copenhagen Psychosocial Questionnaire.

**Figure S1**

***Stress of Conscience as a function Course (ICB vs. ICOP) and Time (Pre, Post, Follow up 1, Follow up 2, Follow up 3)***


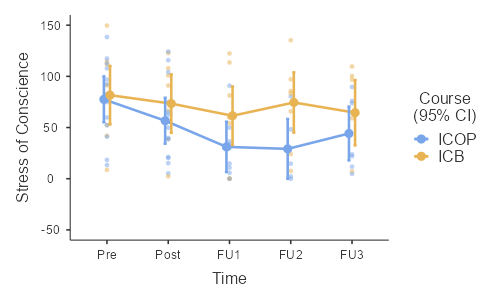


**Figure S2**

***Self-compassion as a function Course (ICB vs. ICOP) and Time (Pre, Post, Follow up 1, Follow up 2, Follow up 3)***


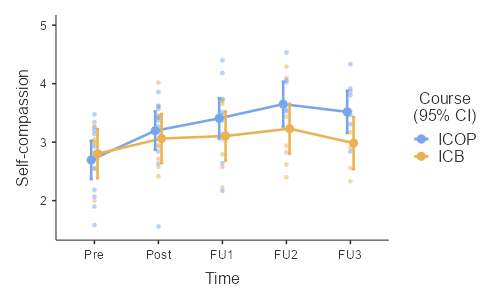


**Figure S3**

***Compassion Satisfaction (PROQOL) as a function Course (ICB vs. ICOP) and Time (Pre, Post, Follow up 1, Follow up 2, Follow up 3)***


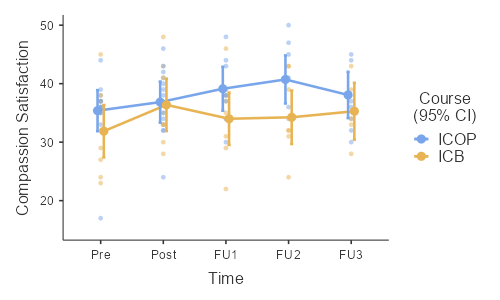


**Figure S4**

***Burnout (PROQOL) as a function Course (ICB vs. ICOP) and Time (Pre, Post, Follow up 1, Follow up 2, Follow up 3)***


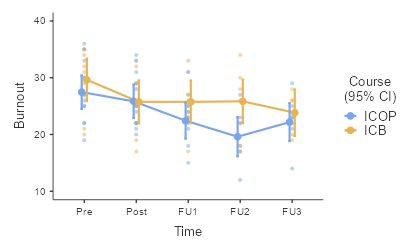


**Figure S5**

***Secondary Traumatic Stress (PROQOL) as a function Course (ICB vs. ICOP) and Time (Pre, Post, Follow up 1, Follow up 2, Follow up 3)***


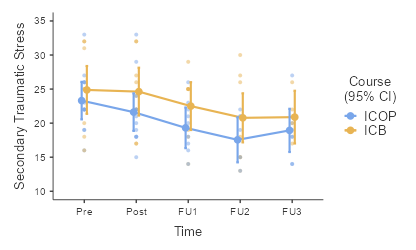


**Figure S6**

***Stress symptoms (COPSOQ) as a function Course (ICB vs. ICOP) and Time (Pre, Post, Follow up 1, Follow up 2, Follow up 3)***


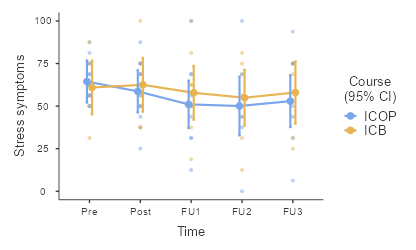


**Figure S7**

***Burnout symptoms (COPSOQ) as a function Course (ICB vs. ICOP) and Time (Pre, Post, Follow up 1, Follow up 2, Follow up 3)***


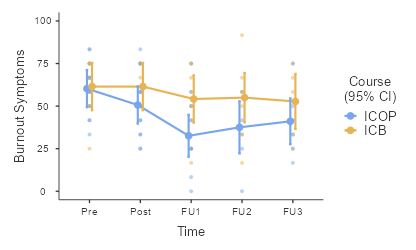


**Figure S8**

***Sleep disturbances (COPSOQ) as a function Course (ICB vs. ICOP) and Time (Pre, Post, Follow up 1, Follow up 2, Follow up 3)***


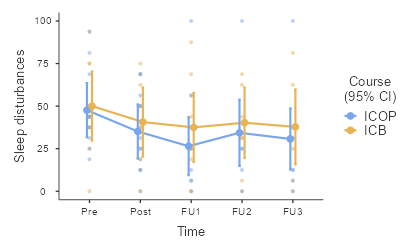

Supplement: Supplementary file 1 — Supplementary material [file mmc1.docx]
